# CTCF promotes colorectal cancer cell proliferation and chemotherapy resistance to 5-FU via the P53-Hedgehog axis

**DOI:** 10.18632/aging.103648

**Published:** 2020-07-20

**Authors:** Qiuhua Lai, Qingyuan Li, Chengcheng He, Yuxin Fang, Simin Lin, Jianqun Cai, Jian Ding, Qian Zhong, Yue Zhang, Changjie Wu, Xinke Wang, Juan He, Yongfeng Liu, Qun Yan, Aimin Li, Side Liu

**Affiliations:** 1 Guangdong Provincial Key Laboratory of Gastroenterology, Department of Gastroenterology, Nanfang Hospital, Southern Medical University, Guangzhou, Guangdong, China

**Keywords:** colorectal cancer, CTCF, Hedgehog, P53, chemotherapy resistance

## Abstract

CTCF is overexpressed in several cancers and plays crucial roles in regulating aggressiveness, but little is known about whether CTCF drives colorectal cancer progression. Here, we identified a tumor-promoting role for CTCF in colorectal cancer. Our study demonstrated that CTCF was upregulated in colorectal cancer specimens compared with adjacent noncancerous colorectal tissues. The overexpression of CTCF promoted colorectal cancer cell proliferation and tumor growth, while the opposite effects were observed in CTCF knockdown cells. Increased GLI1, Shh, PTCH1, and PTCH2 levels were observed in CTCF-overexpressing cells using western blot analyses. CCK-8 and apoptosis assays revealed that 5-fluorouracil chemosensitivity was negatively associated with CTCF expression. Furthermore, we identified that P53 is a direct transcriptional target gene of CTCF in colorectal cancer. Western blot and nuclear extract assays showed that inhibition of P53 can counteract Hedgehog signaling pathway repression induced by CTCF knockdown. In conclusion, we uncovered a crucial role for CTCF regulation that possibly involves the P53-Hedgehog axis and highlighted the clinical utility of colorectal cancer-specific potential therapeutic target as disease progression or clinical response biomarkers.

## INTRODUCTION

Colorectal cancer (CRC) is the third most malignant cancer worldwide and one of the most common tumors of the digestive tract, causing over 600 000 deaths annually [1–4]. Although progress has been made in the development of therapies, including various surgical methods, chemotherapy, radiotherapy and immunotherapy, the prognosis of CRC patients remains unsatisfactory; increasing rates of chemoradiotherapy resistance, local recurrence and distant metastasis result in a poor prognosis among CRC patients [5–9]. A large number of studies have shown that dysregulated genes and the abnormal activation or inhibition of tumor-associated signaling pathways are involved in the initiation and progression of CRC [10–12]. Therefore, we need to gain a more comprehensive understanding of the molecular mechanism involved in the development and progression of CRC and to develop more specific screening tests for the early detection and identification of colorectal tumors with a greater risk of progression.

CTCC-binding factor (CTCF), a transcription factor with 11 zinc fingers (ZFs), is highly conserved despite being over 700 amino acids long [13]. Many intensive studies have reported that CTCF functions as a versatile nuclear factor involved in transcriptional inhibition or activation [14–16], insulation [17, 18], silencers or enhancers [13, 18], gene imprinting [19, 20], controlling X chromosome inactivation in females [21], etc. Most CTCF functions are linked to its ability to regulate three-dimensional (3D) chromatin structure by forming sequence-specific DNA loops [13, 19]. As a multifunctional transcription factor, CTCF was reported to be involved in the initiation of multiple cancers, including breast cancer [18], hepatocellular carcinoma [22], lung cancer [14], prostate cancer [23], etc., which could be attributed to the abnormal expression of CTCF or the dysregulation of its target genes. Some common observations involving CTCF function in cancers include the transcriptional activation of TERT, c-MYC, FOXM1, PLK, GAD1 and other genes [14, 22, 24–26] and the transcriptional repression of p53, BCL6, RASSF1A, CDH1 and others [27–30]. Additionally, chromatin immunoprecipitation (ChIP)-PCR analysis revealed that CTCF affects a number of metastasis-associated genes, including CTBP1, SERPINE1 and SRC [31]. Interestingly, a previous study showed that CTCF has one of the highest mutation rates in CRC [32]. However, the functional role of CTCF in CRC remains unclear.

In the present study, we observed abnormal CTCF expression in CRC. Additionally, we provided the first evidence of CTCF involvement in the P53-Hedgehog signaling pathway and confirmed the effects of aberrant CTCF expression on the cellular biological behavior, including proliferation and chemotherapy resistance to 5-fluorouracil (5-FU), of CRC cells *in vitro* as well as on tumor growth *in vivo*.

## RESULTS

### CTCF is a potential tumor-promoting gene in CRC

**Figure 1:**
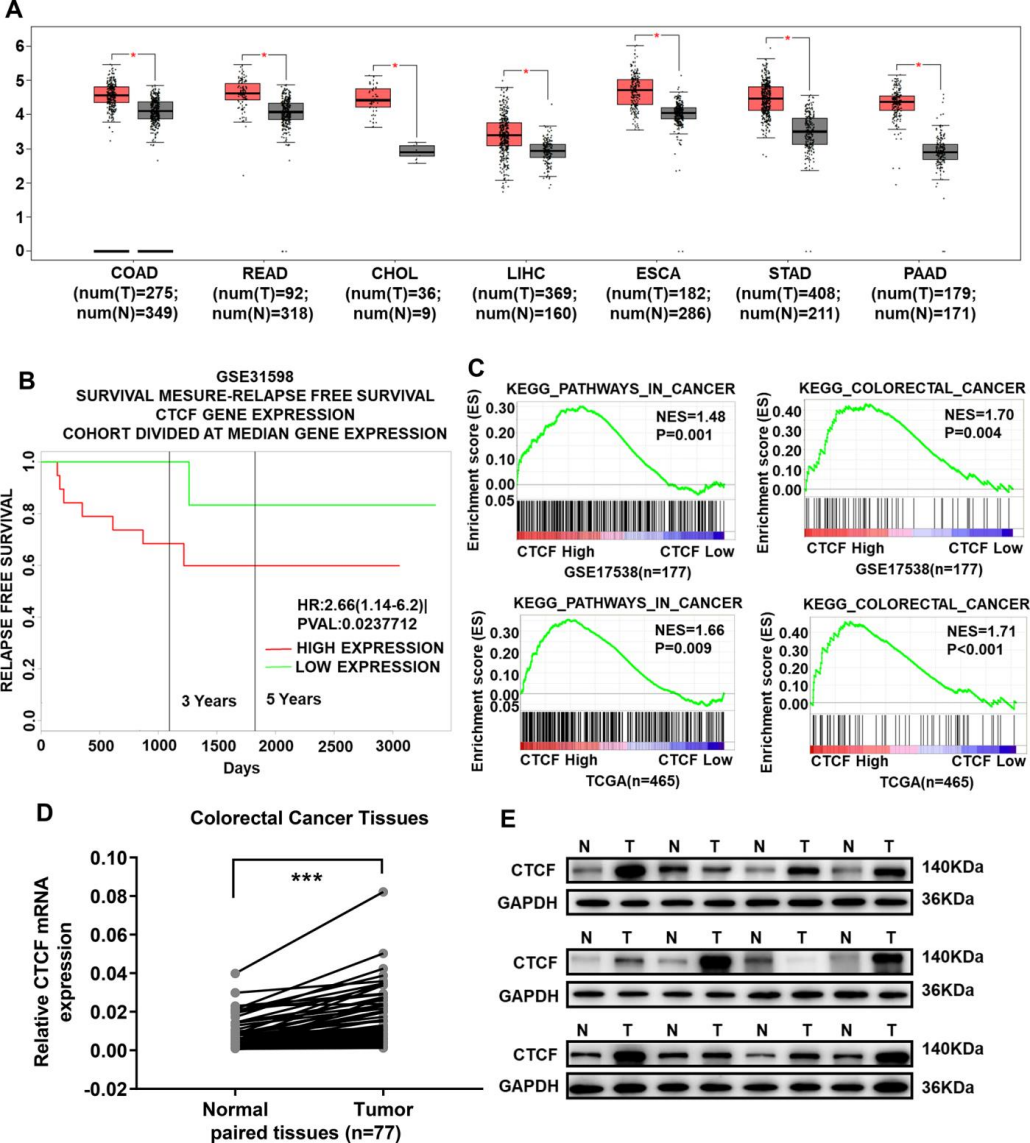
**CTCF is significantly upregulated in CRC tissues and acts as a potential oncogenic gene.** (**A**) CTCF is upregulated in all gastrointestinal tumors (GEPIA, http://gepia.cancer-pku.cn/). (**B**) Kaplan-Meier relapse free survival analysis in CRC patients with high or low expression of CTCF in GSE31598 via online website PROGgeneV2 (http://genomics.jefferson.edu/proggene/). (**C**) GSEA indicated that high expression of CTCF was positively correlated with the cancer related gene set signatures (KEGG_PATHWAYS_IN_CANCER, KEGG_COLORECTAL_CANCER) in CRC patient gene expression profiles (GSE17538, n = 177, and TCGA, n = 465). (**D**) qRT-PCR analysis of CTCF expression in 77 pairs of CRC patient specimens. (**E**) Western blot analyses of CTCF in 12 pairs of tumor and match adjacent normal tissues collected from clinical CRC patients. N for Normal, T for Tumor. The above data are presented as mean ± SEM. * P<0.05, **P<0.01, and ***P<0.001.

An online bioinformatics analysis website, Gene Expression Profiling Interactive Analysis (GEPIA, http://gepia.cancer-pku.cn/) [33], was used to explore the expression of CTCF in The Cancer Genome Atlas (TCGA) database, and the results indicated that CTCF was almost upregulated in all gastrointestinal tumors (Figure 1A). We divided CRC patients into two groups according to CTCF expression levels. Kaplan-Meier (K-M) survival analysis with PROGgeneV2 (http://genomics.jefferson.edu/proggene/) showed that the relapse-free survival time of patients with a high CTCF expression level was significantly shorter than that of patients with a low CTCF expression level in GSE31598 (Figure 1B). Analyses of CTCF-regulated gene set signatures with gene set enrichment analysis (GSEA) indicated that there is a positive correlation between high expression of CTCF and CRC gene set signatures (GSE17538 and TCGA, Figure 1C). Then, we assessed the expression level of CTCF in tumor and paracancerous normal colorectal tissues. CTCF expression in tumor and adjacent normal tissues was analyzed by qRT-PCR and western blot assays (Figure 1D, 1E), and the results showed that CTCF was upregulated in tumor specimens.

To further explore the function of CTCF *in vitro*, we examined CTCF expression in a human embryonic kidney cell line (293T), a human normal colon epithelial cell line (FHC) and six human CRC lines (SW480, SW620, RKO, HCT116 HT29 and LOVO). The expression level of CTCF was relatively high in SW480 cells and was comparatively low in the HCT116 cell line, which was confirmed by both qRT-PCR and western blot analyses (Supplementary Figure 1A, 1B). Herein, we selected SW480 and RKO cell lines to knock down endogenous CTCF expression. On the other hand, HCT116 and RKO cell lines were used to construct cell lines that stably overexpressed CTCF.

### Overexpression of CTCF promotes human CRC cell proliferation

**Figure 2:**
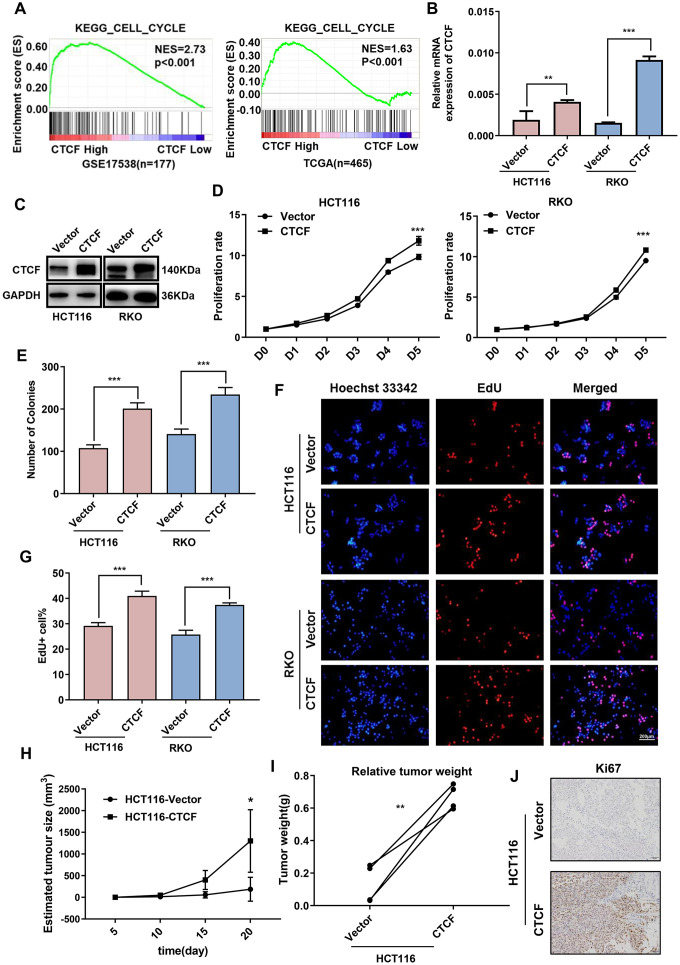
**Upregulation of CTCF promotes human CRC cells proliferation.** (**A**) GSEA plot indicated that high expression of CTCF is positively correlated with the cell cycle gene set signatures (KEGG_CELL_CYCLE) in published CRC patient gene expression profiles (GSE17538, n = 177, and TCGA, n = 465). (**B**, **C**) qRT-PCR and western blot analyses of CTCF expression level in constructed cell lines (HCT116 and RKO).(**D**, **E**) The relative growth rates were measured using CCK8 and colony formation assays and compared between CTCF overexpressed group and Vector group at indicated times in HCT116 and RKO cell lines. (**F**) Images of EdU staining in both indicated cell lines, and the relative percentage of EdU-positive cells in images of related groups are shown (**G**). (**H**, **I**) Tumor volume and weight were measured and analyzed. (**J**) The tumor sections were under IHC staining using antibody against Ki-67. The above data are presented as mean ± SEM. * P<0.05, **P<0.01, and ***P<0.001.

**Figure 3:**
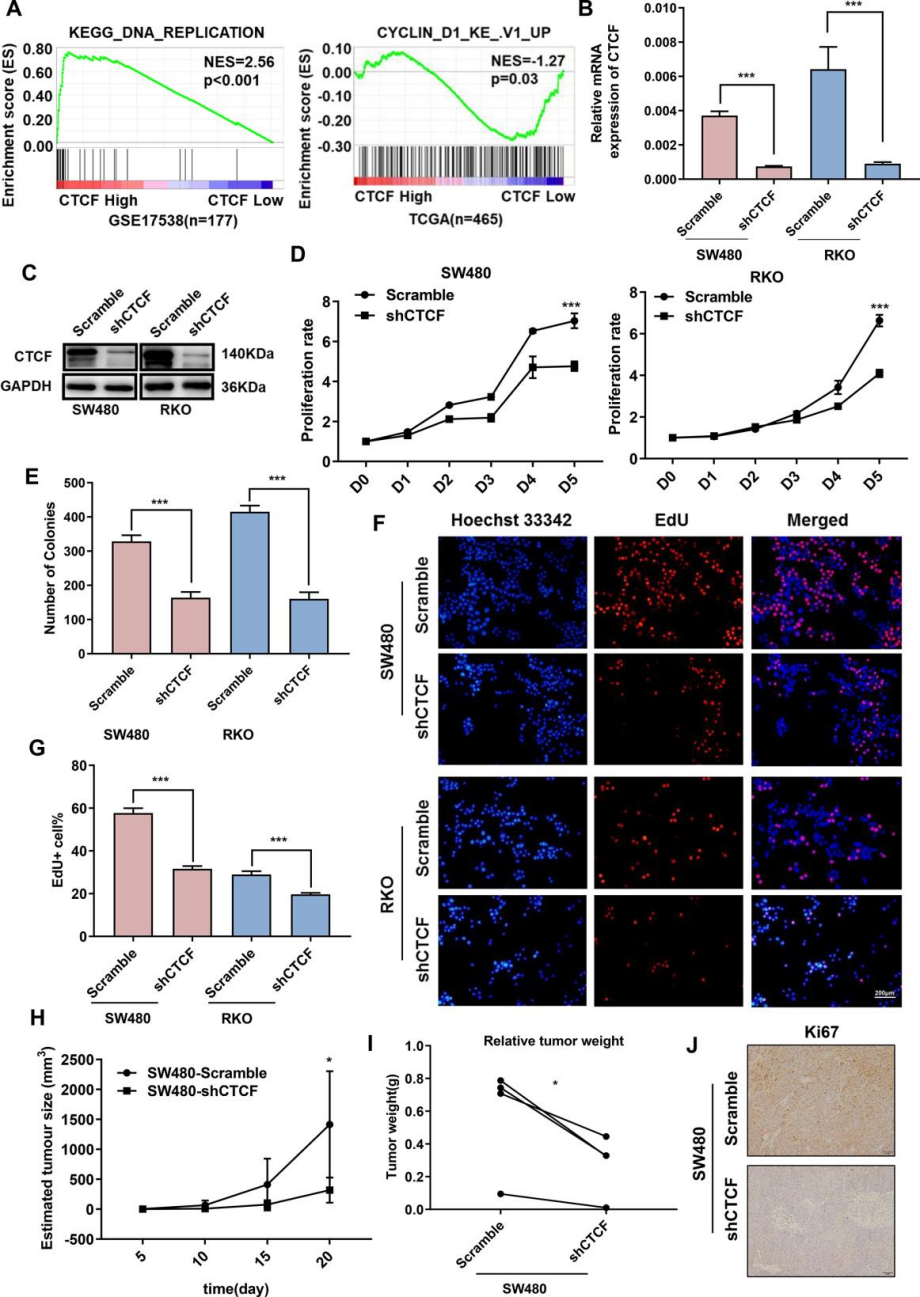
**Inhibition of CTCF represses human CRC cells proliferation.** (**A**) GSEA results showed that “KEGG_DNA_REPLICATION” gene set enriches in the CTCF high expression group and “CYCLIN_D1_KE_V1_UP” gene set enriches in the CTCF low expression group (GSE17538, n = 177, and TCGA, n = 465). (**B**, **C**) qRT-PCR and western blot analyses of CTCF expression level in the constructed cell lines (SW480 and RKO). (**D**–**G**) Cell reproductive capacity was examined by CCK8, colony formation and EdU staining assays. (**H**, **I**) Tumor volume and weight of subcutaneous tumor were measured and analysed. (**J**) Immunohistochemistry was performed to determine Ki-67 expression. The above data are presented as mean ± SEM. * P<0.05, **P<0.01, and ***P<0.001.

As a transcription factor, CTCF has been confirmed to play an essential role in the progression of multiple cancers [13]. To explore the role of CTCF in CRC, GSEA was performed to analyze the relationship between CTCF expression and cell cycle-relevant gene set signatures, and the results revealed that CTCF might promote cell proliferation (Figures 2A, 3A). As mentioned above, we chose two CRC cell lines (HCT-116 and RKO) to construct CTCF-overexpressing cell lines via lentivirus infection. Transfection efficiency was assessed by green fluorescent protein (GFP) (Supplementary Figure 1C). Overexpression effect was confirmed by qRT-PCR (Figure 2B) and western blot (Figure 2C) analyses. CCK-8 and colony formation assays suggested that CTCF upregulation enhanced the proliferative ability in both CRC cell lines (Figure 2D, 2E and Supplementary Figure 1D). In addition, the EdU incorporation assays further confirmed that upregulated CTCF increased the proportion of EdU-positive cells (Figure 2F, 2G).

To investigate whether CTCF is involved in promoting human CRC cell growth *in vivo*, HCT116-CTCF cells and HCT116-Vector cells were subcutaneously injected into the right and left back hips of nude mice (n = 4/group). As shown (Figure 2H, 2I and Supplementary Figure 1E), the tumors in the CTCF-overexpressing group grew more rapidly than the control group tumors. Immunohistochemical (IHC) staining of Ki67 (Figure 2J) further demonstrated that the tumors in the CTCF-overexpressing group displayed much more proliferation than those in the control group.

### Downregulation of CTCF impairs the proliferative capacity of human CRC cells

As previously mentioned, the SW480 cell line, which had the highest CTCF expression level, and RKO cell line were transfected with CTCF-specific shRNA to knockdown endogenous CTCF expression. Similarly, lentiviral infection efficiency was assessed by GFP (Supplementary Figure 2A). Knockdown effect was confirmed by qRT-PCR (Figure 3B) and western blot (Figure 3C) analyses. CCK-8 (Figure 3D), colony formation (Figure 3E and Supplementary Figure 2B) and EdU incorporation assays (Figure 3F, 3G) revealed that the downregulation of endogenous CTCF impaired the proliferative ability in both CRC cell lines. Furthermore, subcutaneous tumorigenesis in nude mice consistently showed that tumors in the SW480-shCTCF group grew much more slowly than the SW480-Scramble group (Figure 3H–3J and Supplementary Figure 2C). Besides, CTCF expression was positively correlated with the expression of CDKs and Cyclins in GEPIA (http://gepia.cancer-pku.cn/, Supplementary Figure 2D).

Thus, these results strongly suggested that CTCF increases the proliferative capacity of CRC cells *in vitro* and in *vivo*.

### CTCF induces chemoresistance in CRC

**Figure 4:**
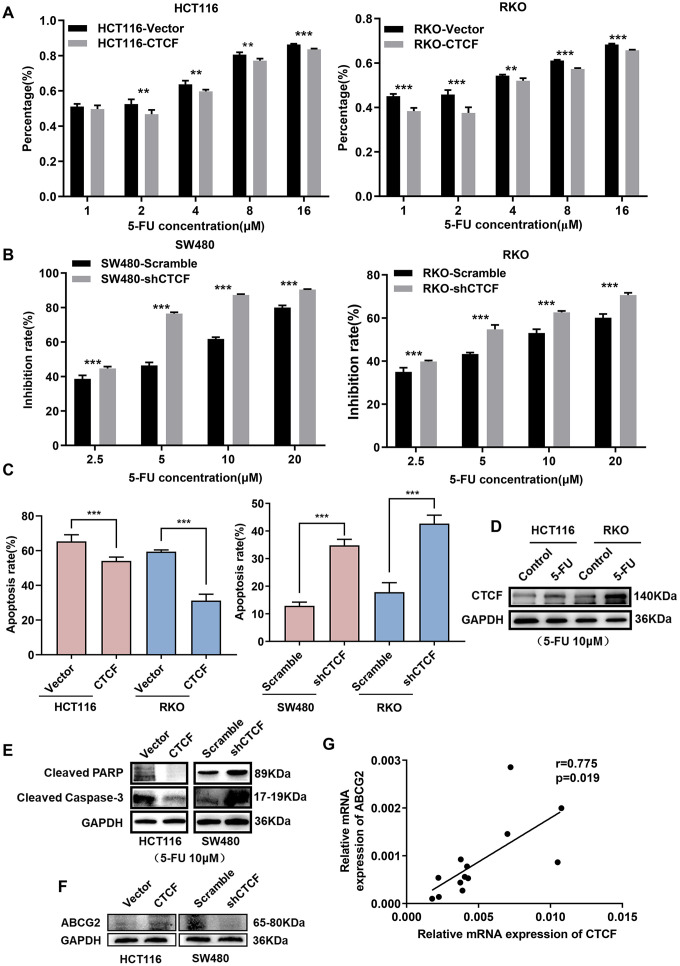
**CTCF induces 5-FU-based chemoresistance in CRC.** (**A**, **B**) Cell growth inhibition rate was measured via CCK8 analysis and compared between different groups with different treatment concentration at indicated time (48 hours). (**C**) The apoptosis rate of different transfected groups with 5-FU treatment were measured by flow cytometry. (**D**) Western blot analysis of CTCF in HCT116 and RKO cell lines after 5-FU (10 μM) treatment for 48 hours. (**E**, **F**) Western blot analyses of cleaved-PARP, cleaved Capase-3 and ABCG2 in HCT116 and SW480 cell lines. (**G**) Spearman correlation analyses between relative CTCF and ABCG2 mRNA expression in 13 fresh human CRC specimens. The above data are presented as mean ± SEM. * P<0.05, **P<0.01, and ***P<0.001.

Furthermore, to explore the possible role of CTCF in chemotherapy resistance, Cell growth inhibition rate was detected after treatment with a concentration gradient of 5-FU, and the results demonstrated that CTCF weakened the cytostatic action of 5-FU (Figure 4A, 4B). Cell apoptosis rate was determined by an apoptosis kit and flow cytometry. The apoptosis rate was increased in the CTCF knockdown group, while it was dramatically decreased in the CTCF-overexpressing group during treatment with 5-FU (Figure 4C and Supplementary Figure 3A, 3B). In addition, we investigated the protein expression level of CTCF and found that CTCF was significantly upregulated after treatment with 5-FU (10 μM) in the HCT116 and RKO cell lines (Figure 4D). Consistently, the expression levels of crucial proteins in the apoptosis pathway were obviously reduced in the CTCF-overexpressing group, while they were increased in the CTCF-knockdown group (Figure 4E).

In further evaluations, the expression of ABCG2, which is a part of the superfamily of ATP-binding cassette (ABC) transporters and plays an important role in the chemotherapy resistance in various tumors [34], was positively related to CTCF expression (Figure 4F). Additionally, clinical specimens further confirmed there was a positive correlation between CTCF and ABCG2 (Figure 4G and Supplementary Figure 3C).

### CTCF enhances malignant behavior in CRC via the Hedgehog signaling pathway

**Figure 5:**
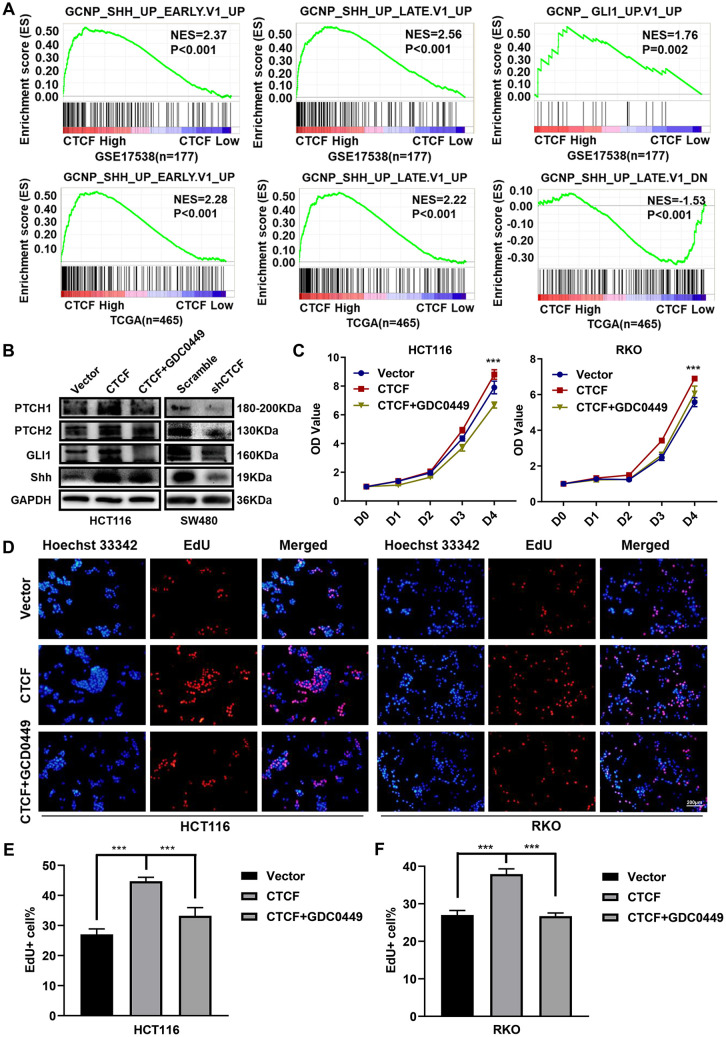
**CTCF activates Hedgehog signaling pathway.** (**A**) GSEA plots indicated that “GCNP_SHH_UP_EARLY.V1_UP” and “GCNP_SHH_UP_LATE.V1_UP” and “GCNP_GLI1_UP.V1_UP” gene signatures enrich in the CTCF high expression group and “GCNP_SHH_UP_LATE.V1_DN” gene set enriches in the CTCF low expression group (GSE17538, n = 177, and TCGA, n = 465). (**B**) Western blot analyses of Key molecules of Hedgehog signaling pathway in different transfected groups with or without the stimulation of Hedgehog signaling pathway inhibitor, GDC-0449 (2 μM). (**C**) Relative growth rate of different transfected groups with or without the administration of GDC-0449 (2 μM). (**D**–**F**) Images of EdU staining of both indicated cell lines with or without the administration of GDC-0449, and the relative percentage of EdU-positive cells in images of related groups is shown. The above data are presented as mean ± SEM. * P<0.05, **P<0.01, and ***P<0.001.

Previous studies reported that Hedgehog signaling pathway activation was closely associated with aggressive phenotypes and chemotherapy resistance in multiple malignancies, including lung cancer [35], pancreatic cancer [36], bladder cancer, etc. [37]. Hence, we examined whether there was a connection between CTCF and the Hedgehog signaling pathway. GSEA was performed to explore CTCF-regulated gene set signatures. The results demonstrated that “GCNP_SHH_UP_EARLY.V1_UP” and “GCNP_SHH_UP_LATE.V1_UP” and “GCNP_GLI1_UP.V1_UP” gene signatures enrich in the CTCF high expression group and “GCNP_SHH_UP_LATE.V1_DN” gene set enriches in the CTCF low expression group (GSE17538 and TCGA, Figure 5A). Then, we carried out western blot assays to investigate whether CTCF can activate Hedgehog signaling pathway in CRC. The results revealed that the expression levels of GLI1, Shh, PTCH1, and PTCH2 were increased in stable CTCF-overexpressing cell lines, while consistent phenomena was observed in the knockdown groups (Figure 5B). Also, correlation analysis in GEPIA revealed that CTCF was positively correlated with GLI1, Shh, PTCH1, and PTCH2 (Supplementary Figure 4A). K-M survival analysis with GEPIA revealed that high expression of GLI1 was accompanied by a shorter overall survival time (Supplementary Figure 4B). Moreover, GDC-0449, a Hedgehog signaling pathway inhibitor, was used for rescue assays, and western blot analyses showed that GDC-0449 counteracted the CTCF-induced activation of the Hedgehog signaling pathway (Figure 5B).

Subsequently, we explored whether the direct blockade of the Hedgehog signaling pathway could restore the function CTCF plays in CRC cell lines. Consistently, we found that treatment with GDC-0449 reversed the enhanced proliferation ability, which was induced by CTCF, based on CCK-8 (Figure 5C) and EdU staining assays (Figure 5D–5F).

### CTCF activates the Hedgehog signaling pathway via transcriptional repression of P53

**Figure 6:**
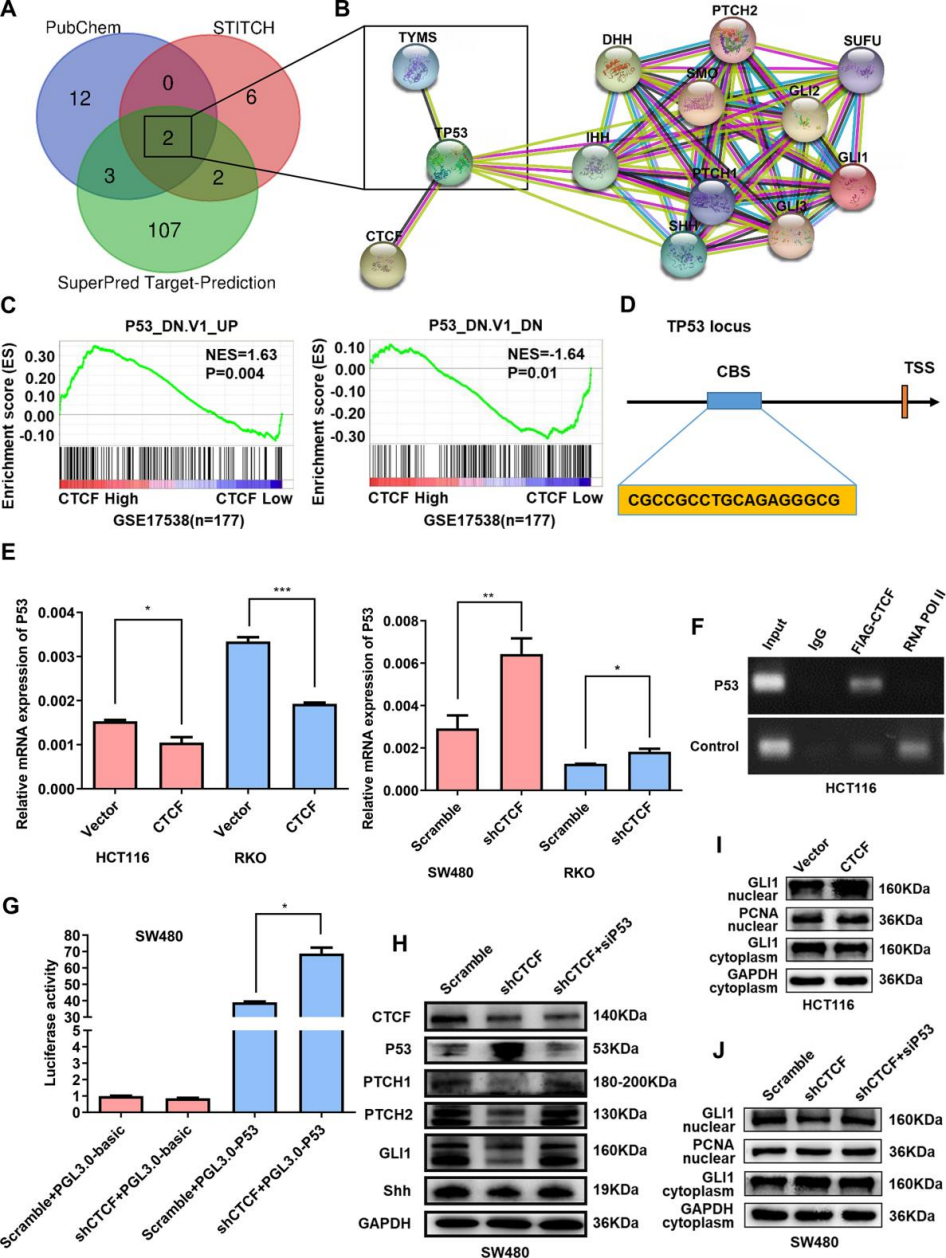
**CTCF enhances Hedgehog signaling pathway activation via targeting P53.** (**A**) Venn diagram enrichment analysis of the 5-FU putative target genes. (**B**) Protein-protein interaction analysis via STRING (https://string-db.org/). (**C**) GSEA plots indicated that “P53_DN.V1_UP” gene set signature enriches in the CTCF high expression group and “P53_DN.V1_DN” gene set enriches in the CTCF low expression group (GSE17538, n = 177). (**D**) Schematic view of the P53 gene transcription start site (TSS) with a CTCF-binding site (CBS). (**E**) qRT-PCR analysis of P53 expression level in constructed cell lines. (**F**) ChIP-PCR results for CTCF on the CBS in HCT116 cells. (**G**) P53 luciferase reporter activity was analyzed in SW480 cells. (**H**) Western blot analysis of Key molecules of Hedgehog signaling pathway and P53 in different transfected groups with or without the stimulation of P53-specific siRNA. (**I**, **J**) Nuclear extract assays and western blot analyses of GLI1 in indicated cells. The above data are presented as mean ± SEM. * P<0.05, **P<0.01, and ***P<0.001.

To predict the potential target genes involved in chemotherapy resistance to 5-FU, 3 bioinformatic target prediction programs (PubChem, STICH, and SuperPred Target-Prediction) were used to explore putative targets of 5-FU. Venn diagram enrichment analysis showed that TYMS and TP53 were theoretical target genes of 5-FU (Figure 6A). Then, we analyzed the interactions among CTCF, the above two target genes and the key molecules of the Hedgehog signaling pathway in functional protein association networks (STRING, https://string-db.org/, Figure 6B), the results suggested that P53 may be a “bridge” between CTCF and the Hedgehog signaling pathway. GSEA plots showed that CTCF was negatively correlated with P53-related gene set signatures (GSE17538, n = 177, Figure 6C). Moreover, GO enrichment were performed to analyze the top 30 similar genes of CTCF in GEPIA (http://gepia.cancer-pku.cn/). As the bubble diagram shown (Supplementary Figure 5A), “p53 binding” was in the top 20 of GO enrichment. qRT-PCR analyses showed that CTCF repressed P53 expression (Figure 6E). K-M survival analysis with GEPIA revealed that high expression of TP53 was accompanied by a longer survival time (Supplementary Figure 5B). Interestingly, high ratio of CTCF/TP53 was accompanied by a shorter disease free survival time while the prognosis of the high TP53/CTCF ratio group was good (Supplementary Figure 5C). A previous study [27] identified CTCF binding site (CBS) in the promoter region approximately 800 bp upstream of the P53 transcription start site (Figure 6D). Therefore, ChIP-qPCR and ChIP-PCR assays were performed to confirm whether CTCF can bind to the site in CRC (Supplementary Figure 6A and Figure 6F). Moreover, a dual luciferase reporter assay showed that the knockdown of CTCF enhanced P53 luciferase activity (Figure 6G). Clinical specimens further confirmed that there was a negative correlation between CTCF and P53 (Supplementary Figure 6B).

To further validate whether CTCF activates the Hedgehog signaling pathway via P53, siRNA targeting P53 was used for rescue assays. Western blot assays demonstrated that the knockdown of P53 counteracted the CTCF knockdown-induced repression of the Hedgehog signaling pathway (Figure 6H). Nuclear extract assays revealed that the inhibition of P53 blocked the CTCF knockdown-induced intranuclear reduction of GLI1 (Figure 6I, 6J). In addition, a colony formation assay showed that the decrease of P53 impaired changes in proliferative capacity caused by CTCF knockdown (Supplementary Figure 6C).

### CTCF-induced chemoresistance is dependent on the P53-Hedgehog axis

**Figure 7:**
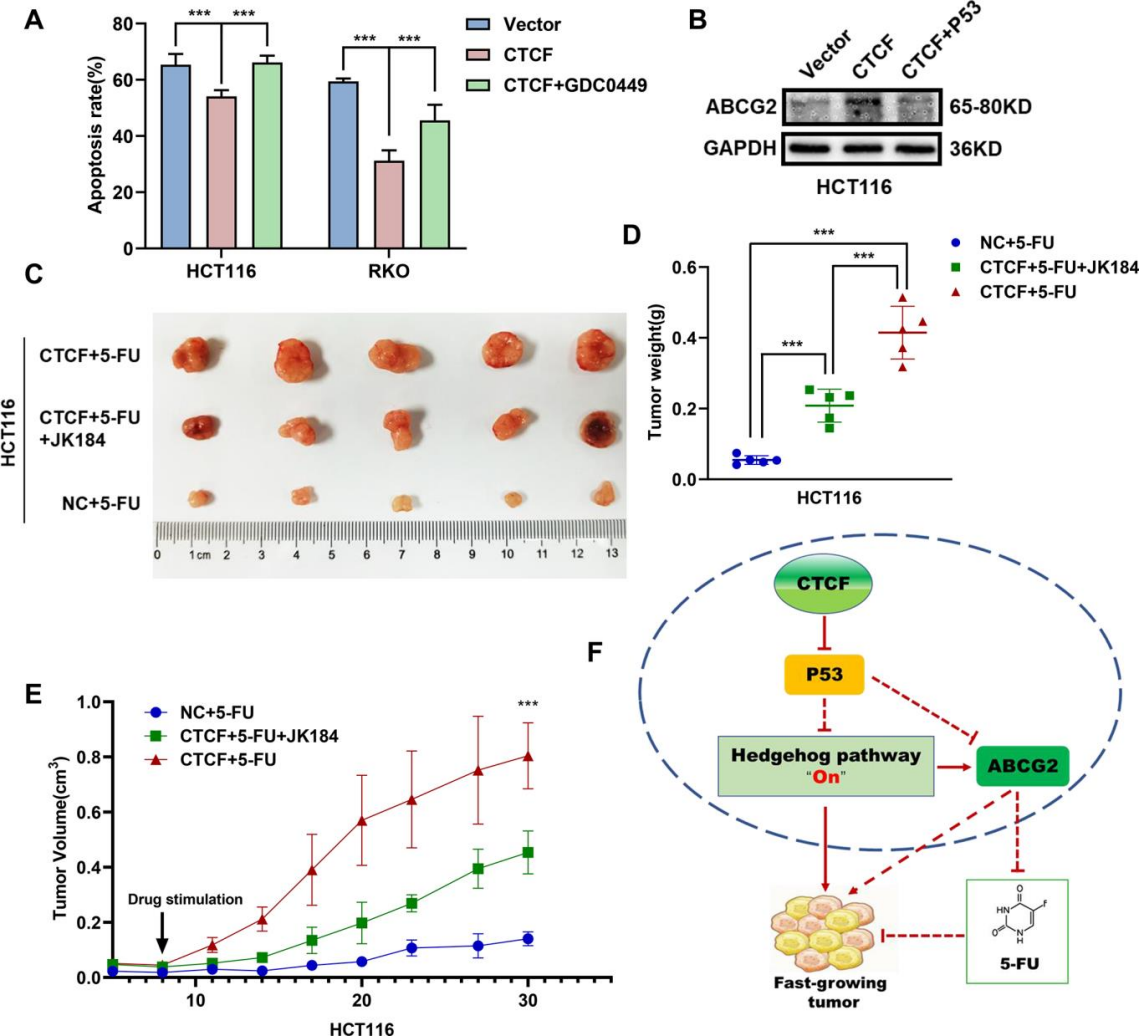
**CTCF promotes chemoresistance by regulating P53-Hedgehog axis signaling.** (**A**) Apoptosis assays showed the effect of GDC0449 on CTCF-mediated 5-FU stimulated apoptosis of CRC cells. (**B**) Western blot analysis of the effects of P53 on CTCF-mediated ABCG2 upregulation. (**C**) The representative images of subcutaneous tumors from different experimental groups are shown. (**D**, **E**) Tumor weight and volume analyses showed that JK184 recovered the stimulative cell proliferation caused by upregulated CTCF under stimulation of 5-FU. (**F**) A hypothetical model illustrating that CTCF transcriptionally represses P53 and activates the Hedgehog signaling pathway to promote proliferation and 5-FU chemotherapy resistance of CRC cells. The above data are presented as mean ± SEM. * P<0.05, **P<0.01, and ***P<0.001.

The above results showed that CTCF can block 5-FU-stimulated cell apoptosis and activate the Hedgehog signaling pathway via transcriptional repression of P53. However, further information is needed to determine whether the associations are causal. Apoptosis assays indicated that administration of GDC-0449 dramatically increased 5-FU-stimulated cell apoptosis rate repressed by overexpression of CTCF (Figure 7A and Supplementary Figure 6D). Western blot analysis revealed that P53 restored the ABCG2 increase induced by CTCF (Figure 7B). Moreover, an *in vivo* tumor growth assay confirmed that the inhibition of the Hedgehog signaling pathway with JK184 recovered the stimulation of cell proliferation caused by upregulated CTCF (n=5/group, Figure 7D, 7E).

## DISCUSSION

CRC is one of the most lethal cancers worldwide. Although progress has been made in CRC diagnosis and treatments, the prognosis of some CRC patients is still poor and is affected by chemotherapy resistance, postoperative recurrence, and metastasis [5, 38].

Studies have revealed that CTCF is robustly upregulated in several cancers and promotes the malignant characteristics of tumor cells [13, 18]. Moreover, Marois Giannakis et al. recently identified recurrently mutated genes in CRC, including TP53, KRAS, SMAD4, CTCF, etc., by performing whole-exome sequencing of 619 incident CRCs and integrating the results with tumor immunity, pathology, and survival data [32]. However, CTCF was not previously studied in CRC progression, which drove us to explore the exact role of CTCF in CRC. Herein, we demonstrated that CTCF was upregulated in CRC tissues by performing western blot and qRT-PCR assays. The overexpression of CTCF promoted malignant phenotypes in CRC by enhancing the proliferative potential and clonogenicity of CRC cells.

Although corresponding 5-FU-based chemotherapies, including FOLFIRI [39], FOLFOX [40], XELOX [41] and other regimens [38], have been developed in the past few years, the clinical outcome remains unsatisfactory, as some CRC patients suffer from chemotherapy resistance. Interestingly, our study indicated that CTCF overexpression reduced the sensitivity of CRC cells to 5-FU and decreased 5-FU-induced apoptosis, which offered a novel explanation for the emergence of chemoresistance in CRC. ABCG2 has been suggested to be involved in clinical multidrug resistance (MDR) in cancer [42], and we found that ABCG2 was activated by CTCF in CRC. Hence, the inhibition of CTCF might be a new strategy to support chemotherapy in CRC patients.

The Hedgehog signaling pathway is involved in many aspects of tumorigenesis and malignant characteristics, including cell cycle progression, proliferation, angiogenesis, migration, invasion, and, in particular, chemotherapy resistance [43, 44]. For example, the persistent activation of the Hedgehog pathway was confirmed to play a critical role in the chemoresistance and prognosis of cancer patients [45]. In addition, ABCG2 is a direct transcriptional target of the Hedgehog signaling pathway and is involved in drug tolerance in diffuse large B-cell lymphoma [46]. Hence, we performed GSEA to analyze the relationship between CTCF and the Hedgehog signaling pathway and found that the enrichment of Hedgehog pathway-related gene set signatures is notably related to CTCF expression. Furthermore, our results showed that the overexpression of CTCF increased GLI1, Shh, PTCH1, and PTCH2 levels, while silencing CTCF induced low GLI1, Shh, PTCH1, and PTCH2 expression. Additionally, the administration of the Hedgehog signaling pathway inhibitor GDC-0449 counteracted the increased proliferation and clonogenicity induced by CTCF upregulation. Furthermore, rescue assays, including *in vivo* tumor growth assays and apoptosis assays, demonstrated that CTCF sustains CRC proliferation and chemotherapy resistance by activating the Hedgehog signaling pathway. However, the mechanism by which CTCF activates the Hedgehog signaling pathway is still unclear.

Thus, the above results indicated that CTCF promotes CRC proliferation and chemotherapy resistance via the Hedgehog signaling pathway. The mechanism by which CTCF activates the Hedgehog signaling pathway needs to be clarified, as previous studies have not reported this relationship. Through network pharmacologic analysis, we found that P53 and TYMS are the potential key target genes of 5-FU. In recent years, more and more studies have shown that TYMS and P53 are crucial molecules in 5-FU resistance [47, 48]. Furthermore, we found P53 might be a key molecule that connects CTCF and the Hedgehog signaling pathway by constructing a protein interaction network. P53 is a crucial molecule in the progression of virtually every malignant tumor [49]. Furthermore, it has been reported that P53 can suppress the Hedgehog signaling pathway [50]. GSEA revealed that CTCF expression is closely related to P53-related gene set signatures. A previous study reported that CTCF transcriptionally inhibits P53 in breast cancer [27]. Hence, we performed qRT-PCR, ChIP and dual luciferase reporter assays to investigate whether the regulatory mechanism between CTCF and P53 also exists in CRC cells. The results showed that CTCF can inhibit P53 transcription via bind to the promoter region of P53.

As mentioned above, studies showed that P53 can suppress the Hedgehog signaling pathway, and our study revealed that CTCF transcriptionally repressed P53 expression. Protein interaction network revealed that P53 is a “bridge” connecting CTCF to Hedgehog signaling pathway. However, whether CTCF activates Hedgehog signaling pathway is P53 dependent in CRC still should be clarified. Hence, we performed rescue assays, including western blot and nuclear extract assays, to investigate the relationship among CTCF, P53 and Hedgehog signaling pathway. Western blot assays demonstrated that CTCF activates the Hedgehog signaling pathway through the repression of P53. Furthermore, nuclear extract assays demonstrated that P53 repression increases the nuclear accumulation of GLI1 which is induced by CTCF. Most of all, subcutaneous xenotransplanted tumor model of human CRC in nude mice further confirmed that CTCF enhanced 5-FU resistance via activating Hedgehog signaling pathway.

In summary, our work provides evidence that CTCF facilitates malignant properties and induces chemotherapy resistance to 5-FU in CRC by regulating the P53-Hedgehog axis. This work introduces a potential biomarker for CRC and a therapeutic target to reduce chemoresistance in patients with CRC.

## MATERIALS AND METHODS

### Clinical specimens and cell culture

With approval from the institutional review board of the hospital ethics committee (Nanfang Hospital, Southern Medical University), clinical CRC specimens and matched normal tissues were collected from 90 patients who underwent surgical treatment for CRC at Nanfang Hospital of Southern Medical University after obtaining informed consent. Additionally, the study was conducted in accordance with the Declaration of Helsinki. CRC was histopathologically confirmed in each patient. Cancer tissues and adjacent normal tissues were frozen at -80°C for storage.

A human embryonic kidney cell line (293T), human normal colon epithelial cell line (FHC) and six human CRC cell lines (SW480, SW620, RKO, HCT116 HT29 and LOVO) were purchased from the Cell Bank of Type Culture Collection (CBTCC, Chinese Academy of Sciences, Shanghai, China) and were cultured in DMEM (Gibco, Carlsbad, CA) supplemented with 10% fetal bovine serum (Gibco, Carlsbad, CA). Cells were maintained at 37°C in a humidified atmosphere with 5% CO2.

### Plasmid construction, lentiviral construction and cell transfection

The overexpression and downregulation of CTCF were achieved by lentiviral delivery. To construct CTCF-overexpressing cell lines, full-length CTCF (NM_006565) was cloned into the expression vector pLenti-EF1a-EGFP-P2A-Puro-CMV (Obio Technology, Shanghai, China) and transfected into HCT116 and RKO cell lines according to the manufacturer’s instructions. The knockdown of CTCF was accomplished with shRNA (Cyagen, Guangzhou, China) that were transfected into SW480 and RKO cell lines according to the manufacturer’s instructions. The CTCF shRNA sequence was sense 5’-GCGAAAGCAGCATTCCTATAT-3’, and the scrambled sequence was sense 5’- CCTAAGGTTAAGTCGCCCTCG-3’. Transduced cells were selected in medium containing puromycin (#EZ2811D376, BioFrox, China) (2 μg/ml) and maintained in medium containing puromycin (1 μg/ml).

### Plasmid and siRNA transfection

To exogenously overexpress TP53, full-length TP53 (NM_000546.6) was cloned into the expression vector pENTER (Vigene, Shandong, China). The knockdown of TP53 was achieved by siRNA (Genecopoeia, Shanghai, China). The TP53 siRNA sequence was sense 5’-GAAGAAACCACUGGAUGGATT -3’, and the negative control sequence was sense 5’-UUCUCCGAACGUGUCACGUTT-3’. Genomic DNA fragments from the TP53 locus (-1000-0 bp of the TP53 promoter region) were cloned into the pGL3-basic vector (Obio Technology, China). Plasmids or siRNA were transfected into CRC cells with LipofectamineTM 3000 (Invitrogen, Carlsbad, CA) according to the manufacturer's instructions.

### Cell Counting Kit-8 (CCK-8), colony formation assays

CCK-8 (Dojindo, Japan) analysis was used to estimate cell proliferation. The transfected cell lines were cultured on the 96-well plates and then with culture medium containing 10ul CCK-8 each well and incubated for 2 hours. Proliferation was determined by absorbance measurement at 450 nm using a microplate reader.

Cells were seeded in 6-well plates at a density of 5 ×10^2^ per well and incubated at 37 °C in a humidified atmosphere with 5% CO2 and the medium was replaced every 3-4 days. The colonies were counted and analysed in about two weeks. The experiment was performed with at least three replicates for each cell line.

### 5-Ethynyl-2’-deoxyuridine (EdU) incorporation and apoptosis assays

As the manufacturer’s instructions for a Cell-Light EdU DNA cell proliferation kit (#C103010-1, RiboBio, China) described, transfected cell lines were seeded in 96-well plates and incubated with EdU in medium (50 μM) for 2 hours. Then, cells were washed twice and fixed in 4% paraformaldehyde for 10 min, permeabilized with 0.5% Triton-X 100, and stained with Apollo® fluorescent dye. Photographs of cells were independently taken with an OLYMPUS confocal microscope.

The cells were analyzed by FACS according to the standard protocol provided by the manufacturer (BD FACSAria II). Apoptosis was measured by using an Annexin V-PE/7-Amino-Actinomycin (7-AAD) Apoptosis Detection Kit (#559763, BD Biosciences Pharmingen, US). After treatment with 5-FU (#9648, TargetMol, China) at the indicated concentrations for 48 hours, cells were harvested by trypsinization, washed with cold PBS and then resuspended in 1X binding buffer. Then, 5 μl of PE Annexin V and 5 μl 7-AAD were added to each tube. The suspension was then mixed well and incubated for 15 min in the dark at room temperature (RT) (25°C). After resuspension, samples were analyzed by flow cytometry.

### RNA isolation, cDNA synthesis, and quantitative real-time PCR (qRT-PCR)

Total RNAs were extracted from cells or fresh surgical CRC tissues with Trizol solution (TaKaRa). Quantitative real-time polymerase chain reaction (qRT-PCR) was performed in triplicate using the PrimeScript RT Reagent Kit, SYBR Premix Ex Taq (TaKaRa) and a Roche Light Cycler 480 Real-Time PCR System (Roche Diagnostics, Mannheim, Germany) following the manufacturer’s instructions. Relative gene expression levels were normalized to the expression of GAPDH. qRT-PCR results were analyzed to obtain Ct values of amplified products, and data was analyzed by the 2-ΔΔCt method. The specific primers used for detection are listed in Supplementary Table 1.

### Western blot and immunohistochemistry (IHC)

We performed western blot according to the previous study [51]. Protein lysates were prepared, subjected to SDS-PAGE, transferred onto PVDF membranes and blotted according to standard methods by using following antibodies: CTCF(#2899, Cell Signaling), PTCH1 (#2648, Cell Signaling), and PTCH2 (#2470, Cell Signaling), GLI1(#3538, Cell Signaling), Shh(#2207, Cell Signaling), GAPDH (60004-1-Ig, Proteintech), Cleaved PARP (BF9106, Affinity), Cleaved Caspase-3 (# 9661S, Cell Signaling), TP53 (AF0879, Affinity), ABCG2 (#42078T, Cell Signaling).

Immunohistochemistry was performed following the manufacturer’s instructions (PV-6001, ZSGB-BIO, Beijing, China) and used the antibody (anti-Ki67, 27309-1-AP, Proteintech)

### ChIP and dual luciferase reporter assays

ChIP assays were performed with a kit (#17–10085, Merck) as previously described [51]. The CTCF binding site (CBS) at the transcriptional start site of TP53 was amplified with qRT-PCR and PCR. The specific primers are included in Supplementary Table 1. Luciferase activity was detected with a dual luciferase assay kit (Promega, America), as previously described [52].

### Network pharmacologic analysis

List of websites used as follows, PubChem: https://pubchem.ncbi.nlm.nih.gov/, STITCH: http://stitch.embl.de/, SuperPred Target-Prediction: http://prediction.charite.de/index.php?site=chemdoodle_search_target, STRING: https://string-db.org/. The Canonical SMILES of 5-FU is C1=C(C(=O)NC(=O)N1)F, and the InChI Key of 5-FU is GHASVSINZRGABV-UHFFFAOYSA-N.

### *In vivo* experiments

Female athymic 4- to 5-week-old Balb/C (nu/nu) mice were purchased from the Laboratory Animal Services Centre of Guangdong Province and were maintained in a specific pathogen-free facility. For the tumor growth assay, 5 × 10^6^ cells were subcutaneously injected into the right and left back of nude mice (n =4/group). For drug treatment assays, 1×10^7^ cells were subcutaneously injected into the right back of nude mice (n =5/group). JK184 (5 mg/kg body weight, #315703-52-7, MCE) and 5-FU (23 mg/kg body weight, #51-21-8, MCE) were used to treat nude mice according to the manufacturer’s instructions. The tumor volume was calculated using the following formula: V = 0.5 × D × d2 (V represents volume, D represents the longitudinal diameter, and d represents the latitudinal diameter). The use of animals was approved by the Nanfang Hospital Animal Ethics Committee (ethical code NFYY-2018-38; approval date-10 April 2018).

### Statistical analysis

All experiments were performed at least thrice. The SPSS 17.0 (SPSS; Chicago, IL, USA) statistical analysis software was used for statistical analysis of experimental data. The significance of differences between groups was estimated by Student’s t-test. Additionally, multiple group comparisons were analyzed with one-way ANOVA. * P<0.05, **P<0.01, and ***P<0.001 were considered significant.

## Supplementary Material

Supplementary Figures

Supplementary Table 1
