# Supplementary material for: CTCF promotes colorectal cancer cell proliferation and chemotherapy resistance to 5-FU via the P53-Hedgehog axis
Source: Aging (Albany NY). 2020 Jul 20;12(16):16270–93. doi: 10.18632/aging.103648 (PMC7485712; doi:10.18632/aging.103648)
Supplement: Supplementary Figures [file aging-12-103648-s002..pdf]

SUPPLEMENTARY FIGURES

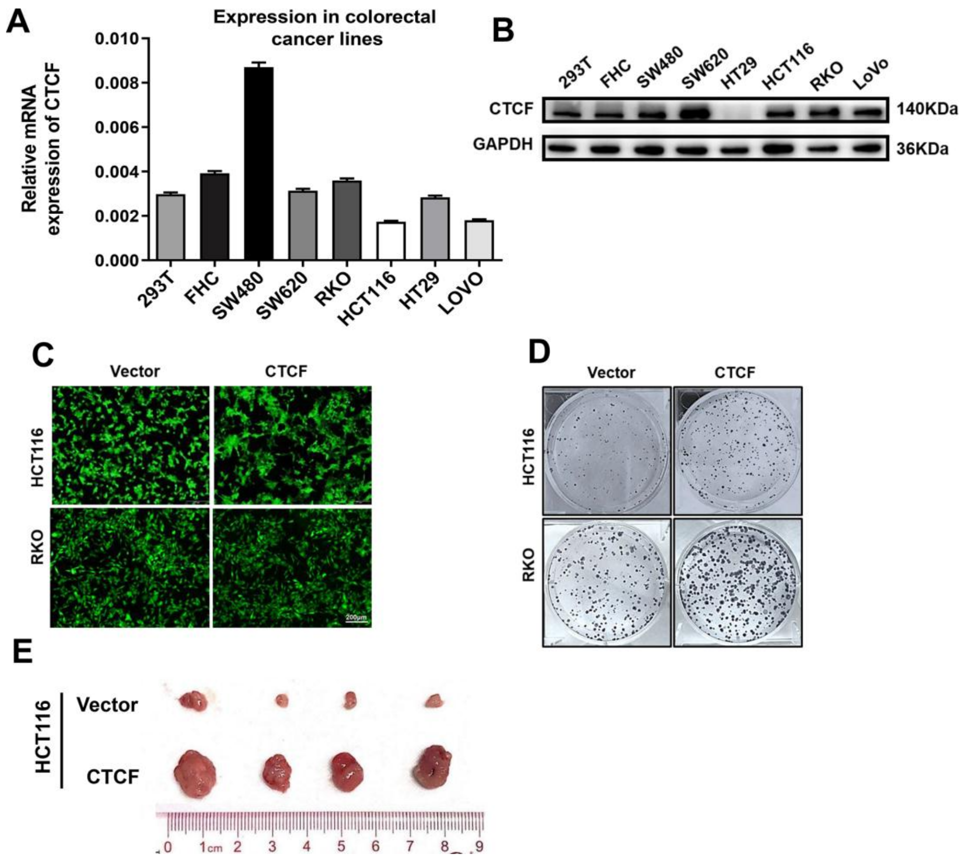

Supplementary Figure 1. Overexpression of CTCF enhances human CRC cells proliferative capacity. (**A**) mRNA level of CTCF in human embryonic kidney cell line (293T), human normal colon epithelial cell line (FHC) and six CRC cell lines. (**B**) Western blot analysis of human embryonic kidney cell line (293T), human normal colon epithelial cell line (FHC) and six CRC cell lines. The above data are presented as mean ± SEM. (**C**) Fluorescence assessment of the green fluorescent protein (GFP). Vector represents lentivirus-mediated control groups. CTCF represents lentivirus-mediated CTCF overexpressing groups. (**D**) The representative images of colony formation assay from different experimental groups are shown. (**E**) Corresponding cells were respectively injected subcutaneously into the right and left back hips of nude mice (n = 4). Representative images of the tumors are shown. * P<0.05, **P<0.01, and ***P<0.001.

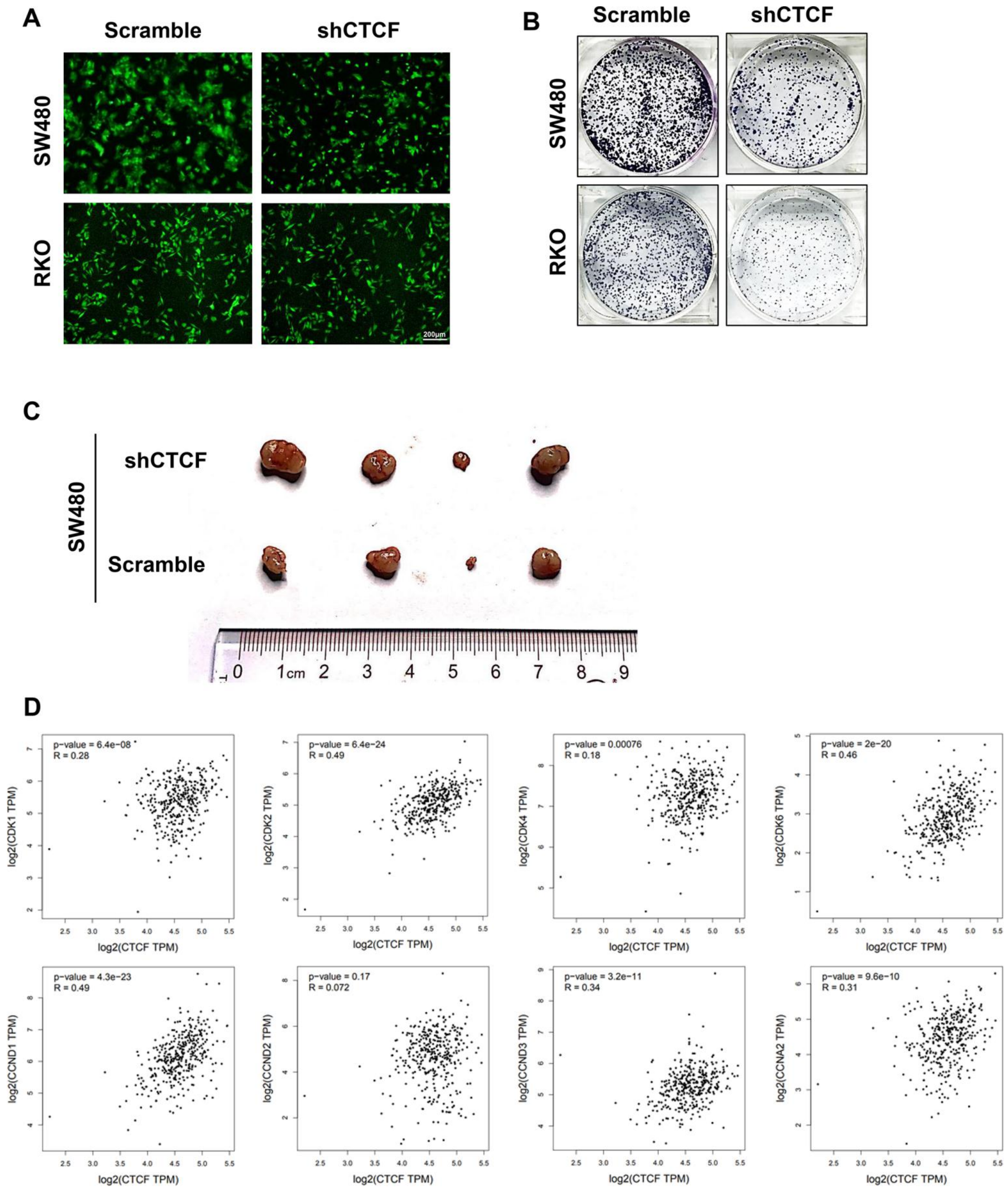

Supplementary Figure 2. Knockdown of CTCF inhibits human CRC cells proliferation. (**A**) Fluorescence assessment of the green fluorescent protein (GFP). Scramble represents lentivirus-mediated control groups. shCTCF represents lentivirus-mediated CTCF silencing groups. (**B**) The representative images of colony formation assays from different experimental groups are shown. (**C**) Corresponding cells were respectively injected subcutaneously into the right and left back hips of nude mice (n = 4). Representative images of the tumors are shown. (**D**) CTCF expression was positively correlated with the expression of CDKs and Cyclins in GEPIA (http://gepia.cancer-pku.cn/).

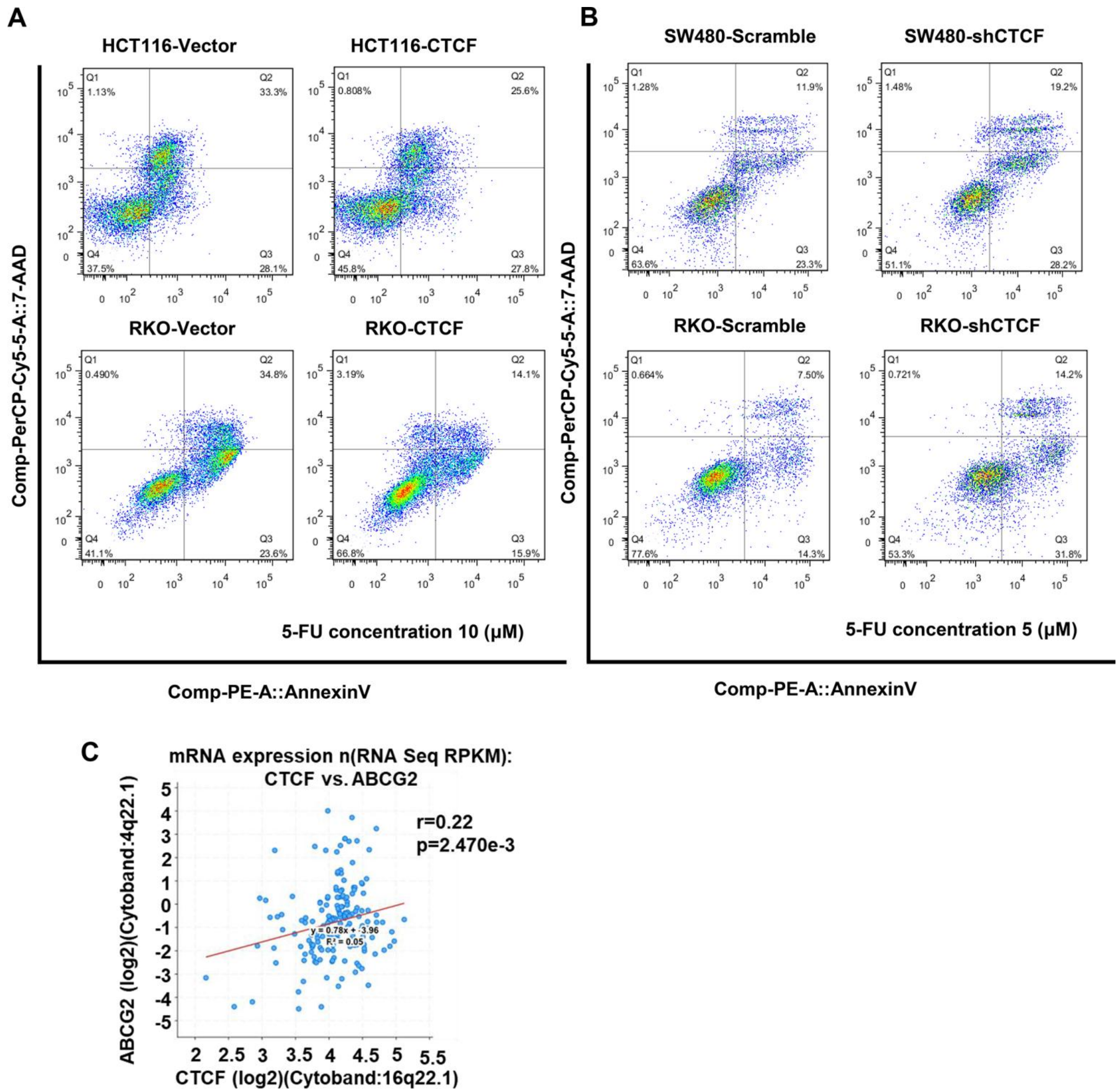

Supplementary Figure 3. CTCF causes CRC cells to be insensitive to 5-FU-based chemoresistance. (**A**, **B**) The representative images of apoptotic assays from different experimental groups are shown. Scramble and shCTCF cell lines were treated with 5-FU with a 5μM concentration, and the other cell lines were treated with 5-FU with a 10μM concentration. (**C**) Positive correlation between CTCF expression and ABCG2 by spearman correlation analysis in cBioPortal (http://www.cbioportal.org/).

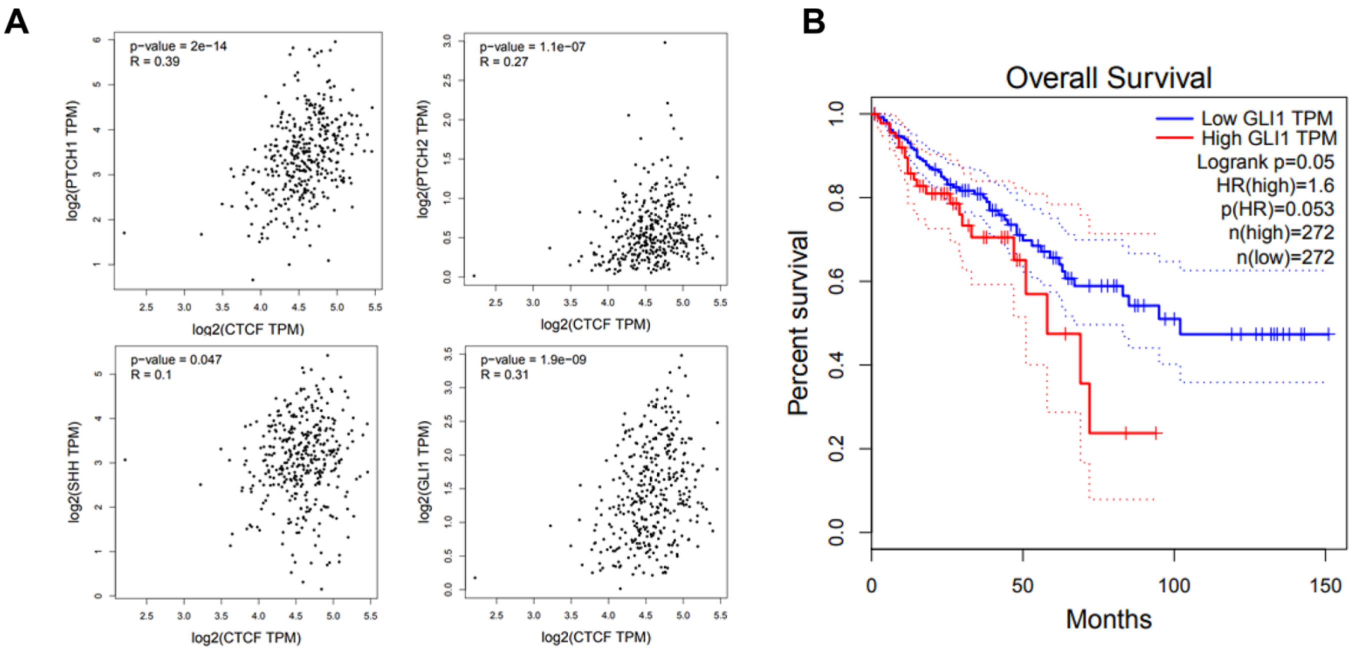

Supplementary Figure 4. CTCF activates Hedgehog signaling pathway. (**A**) Positive correlation between CTCF expression and the key molecules of Hedgehog signaling pathway by spearman correlation analsis in GEPIA (http://gepia.cancer-pku.cn/). (**B**) Kaplan-Meier survival analysis revealed that CRC patients in the GLI1 high expression group have shorter survival time in GEPIA.

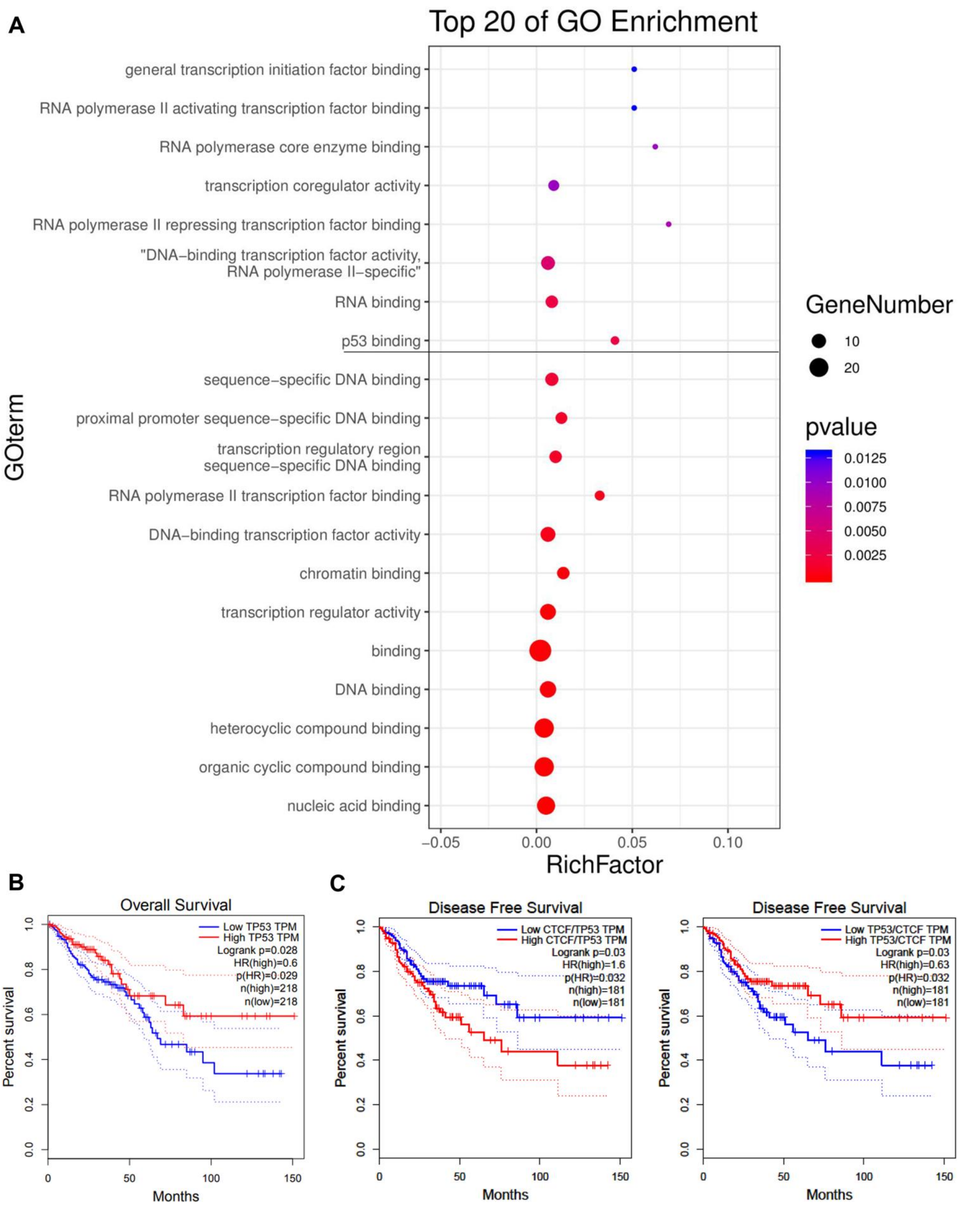

Supplementary Figure 5. CTCF might regulate P53 in CRC. (**A**) GO-molecular function enrichment analysis of the top 30 similar genes of CTCF in GEPIA is shown. (**B**) Kaplan-Meier survival analysis revealed that CRC patients in the TP53 high expression group have good prognosis in GEPIA. (**C**) Kaplan-Meier survival analysis showed that the ratio of CTCF/TP53 is positively related to disease free survival time.

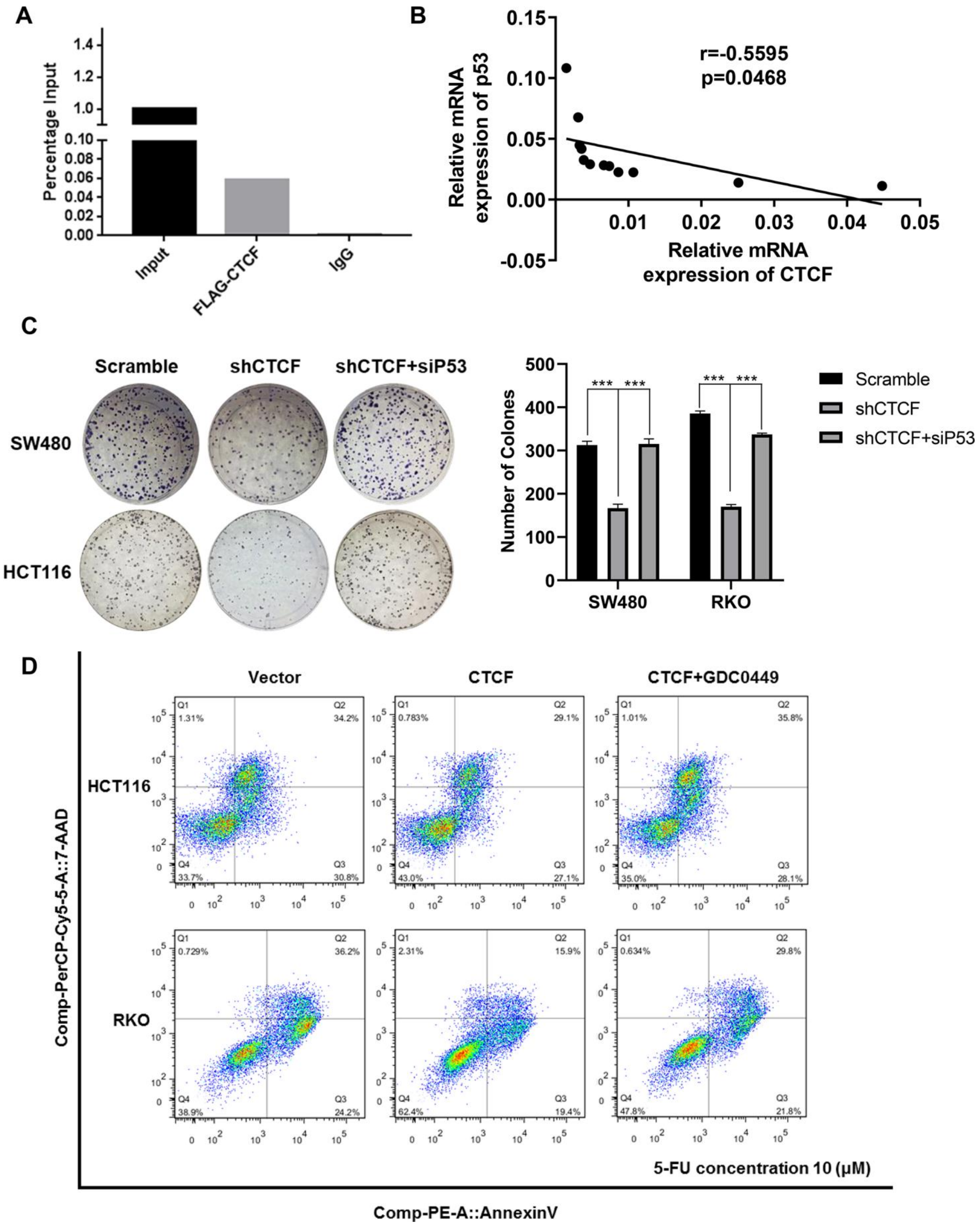

Supplementary Figure 6. CTCF facilitates CRC progression via P53-Hedgehog axis. (**A**) ChIP-qPCR results for CTCF on the CBS in HCT116 cells. (**B**) Spearman correlation analysis between relative CTCF and P53 mRNA expression in 13 fresh human CRC specimens. (**C**) Cell proliferative capacity was tested by colony formation assays and the representative images of colony formation assays from different experimental groups are shown. (**D**) The representative images of apoptotic assays from different experimental groups with or without administration of GDC-0449 are shown. The above data are presented as mean ± SEM. * $P<0.05$, ** $P<0.01$, and *** $P<0.001$.
