# Supplementary material for: CTCF promotes colorectal cancer cell proliferation and chemotherapy resistance to 5-FU via the P53-Hedgehog axis
Source: Aging (Albany NY). 2020 Jul 20;12(16):16270–93. doi: 10.18632/aging.103648 (PMC7485712; doi:10.18632/aging.103648)
Supplement: Supplementary Table 1 [file aging-12-103648-s001..pdf]

SUPPLEMENTARY TABLE

Supplementary Table 1. Primer sequences used for real-time PCR or ChIP-PCR (5′ to 3′).

Gene	Forward primer	Reverse primer
GAPDH	CGAGCCACATCGCTCAGACA	GTGGTGAAGACGCCAGTGGA
CTCF	CCCAAACAGAACCAGCCAAC	TCCTCTTCCTCTCCCTCTGC
ABCG2	CTCTTCTTCCTGACGACCAACCA	ATGACACTCTGTAGTATCCGCTGAT
TP53	CAGCACATGACGGAGGTTGT	TCATCCAAATACTCCACACGC
CTCF-CBS	CATTGTTGTATTCCTGAGTGCC	GAGTCCCGCGGTAAT TCTT
